# Epigenetic and genetic dispositions of ovarian carcinomas

**DOI:** 10.18632/oncoscience.82

**Published:** 2014-09-22

**Authors:** Ken Yamaguchi, Noriomi Matsumura, Masaki Mandai, Tsukasa Baba, Ikuo Konishi, Susan K Murphy

**Affiliations:** ^1^ Department of Gynecology and Obstetrics, Graduate School of Medicine, Kyoto University, Kyoto, 606-8507 Japan; ^2^ Department of Obstetrics and Gynecology, Kinki University, Faculty of Medicine, Osakasayama, Osaka, 589-8511 Japan; ^3^ Department of Obstetrics and Gynecology, Duke University Medical Center, Durham NC, 27708 USA

**Keywords:** epigenetics, methylation, high-grade serous adenocarcinoma, ovarian clear cell carcinoma

## Abstract

Ovarian clear cell carcinoma has unique clinical characteristics with slow growth and a stress-resistant phenotype that is epigenetically induced during cancer progression in an inflammatory microenvironment. We refer to this as an epigenetic disposition, which is frequently associated with unique biomolecular features including prominent alterations in methylation, microsatellite instability and *ARID1A* mutations. This characteristic methylation profile also affects glucose metabolism, commonly known as the Warburg effect. In contrast, high-grade ovarian serous adenocarcinoma has a genetic disposition that is accompanied by rapid growth, *TP53* mutations and chromosomal instability. The concept of epigenetic and genetic dispositions is applicable to various malignancies, including gastric and colorectal cancers. These disposition classifications are based on fundamental characteristics of malignancies and may provide a new vantage point for development of individualized therapies.

## INTRODUCTION

Ovarian cancer has the worst mortality of all malignant gynecologic diseases. Improved understanding of the heterogeneous features of this disease, including distinct clinicopathological and molecular characteristics, are needed to develop individualized therapeutic strategies [1]. The most frequent histological subtype among epithelial ovarian cancers is high-grade serous adenocarcinoma (SAC). SAC develops *de novo* from the fallopian tube epithelium [2]. The majority of SACs exhibit rapid proliferation and are at an advanced stage at the time of diagnosis. Following surgical debulking and chemotherapy, most show a favorable response. *TP53* mutations are present in 96% of SACs, leading to chromosomal instability [3, 4]. In contrast, ovarian clear cell carcinoma (CCC) has distinct clinical and biomolecular features as compared to the other subtypes of ovarian cancer. Properties of ovarian CCC include development associated with endometriosis, chemotherapeutic resistance and thromboembolism complications [5]. These characteristics, referred to as “ovarian CCC-likeness”, are affected in the carcinogenic process by a stressful inflammatory environment [6]. Mutations of *ARID1A*, a member of the SWI/SNF chromatin remodeling complex, occur in approximately half of ovarian CCC [6-8], and overexpression of HNF1B is a hallmark of ovarian CCC [5, 9]. Epigenetically, DNA methylation profiles of ovarian CCC also differ substantially from that of the other subtypes [10]. We hypothesize here that different histologic subtypes of ovarian cancer exhibit epigenetic or genetic dispositions. Several other cancers, including gastric and colon cancers, also show distinctive dispositions and develop in the same organs as certain subtypes of ovarian cancer. Elucidation of these dispositions will lead to advancement of novel biomarker development and help advance implementation of individualized therapies. In this article, we describe the clinical and biomolecular differences that define epigenetic and genetic dispositions in ovarian cancer, and how these might apply to other malignancies.

### *Epigenetic* and *genetic dispositions* of ovarian cancer

**Figure 1:**
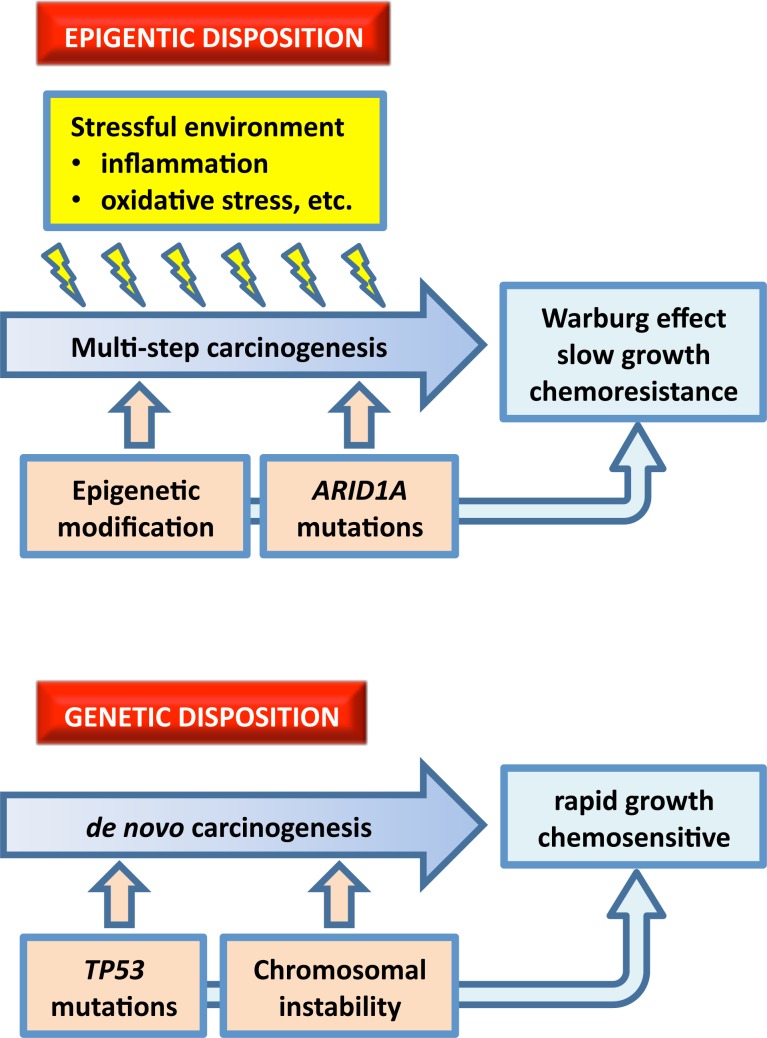
Overview of the features that contribute to epigenetic and genetic dispositions An epigenetic disposition develops in a stressful microenvironmental context, whereas a genetic disposition is dominated by *TP53* mutations and chromosomal instability. An epigenetic disposition is associated with *ARID1A* mutations and the Warburg effect, which is also epigenetically regulated.

Recent genome-wide technologies have allowed us to group malignancies based on genetic and epigenetic classifications. Ovarian SAC is initiated by *TP53* mutations whereas *ARID1A* mutations are observed in the development of roughly half of ovarian CCC [3, 8]. In ovarian CCC, ARID1A and HNF1B have different functional roles. Mutations of tumor suppressor *ARID1A* contributes to carcinogenesis in several cancers, including ovarian cancer [11]. HNF1B function is central to the definition of the fundamental ovarian CCC-likeness characteristics. HNF1B influences thrombosis and glucose metabolism, in particular for cancer cells exhibiting the Warburg effect [12, 13]. The expression of *HNF1B* is epigenetically regulated by DNA methylation. Indeed, ovarian CCCs have unique methylation profiles that distinguish them from other histological subtypes of ovarian cancer [10]. In CCC, HNF1 pathway genes are activated by coordinate hypomethylation, while many genes belonging to the estrogen receptor (ER) network are suppressed by synchronous hypermethylation. Alterations in DNA methylation are thought to be an early event in cancer progression [14]. Genetic inactivation of *ARID1A* initiates carcinogenesis while epigenetic alterations contribute to biological phenotypes as well as progression of ovarian CCC. On the other hand, *TP53* mutations initiate carcinogenesis and also contribute to the rapid growth that characterizes ovarian SAC. In terms of biomolecular features, SAC with *TP53* mutations are dominated by copy number alterations [4]. To help distinguish these biological characteristics, we refer to SAC as having a genetic disposition while CCC has an epigenetic disposition (Figure 1).

The biological features of several malignancies arising in other organs lends itself to their classification as having an epigenetic or genetic disposition. Aberrant DNA methylation is frequently observed in cancers associated with chronic inflammation and infection with viruses or other pathogenic microorganisms, such as Epstein– Barr virus, *Helicobacter pylori*, hepatitis B or C viruses or human papilloma virus. Genome-wide assessments have led to the identification of novel genetic mutations and epigenetic subclassifications. Kai et. al. showed hypermethylation, representing an epigenetic disposition, is observed in microsatellite instability (MSI) and Epstein- Barr virus (EBV) subgroups of gastric cancer, but not in intestinal and diffuse subtypes of gastric tumors. Intestinal and diffuse subgroups frequently exhibit *TP53* mutations (56%) without increased DNA methylation, conforming to a genetic disposition of gastric cancer [15, 16]. The diffuse subtype of gastric cancer involves chromosomal alterations similar to ovarian SAC [17]. Alteration of DNA methylation is an earlier event, preceding chromosomal instability during hepatocarcinogenesis with chronic inflammation and/or persistent viral infection [18]. Some colorectal cancers have a high frequency of DNA methylation in specific CpG islands, referred to as the “CpG island methylator phenotype” (CIMP) [19]. Epigenetic profiling identified three DNA methylation- based subgroups of colorectal cancer: CIMP-high, CIMP- low and CIMP-negative [19-22]. The CIMP-high subgroup is associated with MSI (80%) and BRAF mutations (53–100%) as well as rare KRAS and TP53 mutations (0–18% and 11%, respectively), indicating an epigenetic disposition. The CIMP-low subgroup is characterized by a high rate of KRAS mutations (45–92%) and lower rates of MSI, BRAF, or TP53 mutations (0, 0–4, and 31–38% respectively), suggesting a mixed genetic and epigenetic disposition. CIMP-negative cases have a high frequency of TP53 mutations (71–74%), copy-number alterations [23] and rare MSI (12%) or mutations of BRAF (0–2%) or KRAS (9–33%), representing the genetic disposition subgroup in colorectal cancer. Together these findings suggest that an epigenetic disposition may be involved in some inflammation-induced cancers, while subtypes that are overrepresented by TP53 mutations demonstrate a genetic disposition. These biomolecular features are summarized in Table 1.

**Table 1:** Epigenetic and genetic dispositions of malignancies from different organs

Cancer	Subtypes	Molecular features
**Ovarian cancer**	**Epigenetic disposition** Clear cell carcinoma	*ARID1A* mutations (46–57%) *HNF1B* hypomethylation ER pathway hypermethylation
	**Genetic disposition** High-grade serous adenocarcinoma	*TP53* mutations (96%) Chromosomal instability
**Gastric cancer**	**Epigenetic disposition** Microsatellite instability (MSI) type	Hypermethylation MSI *ARID1A* mutations (83%)
	Epstein-Barr virus (EBV) type	Hypermethylation *ARID1A* mutations (73%)
	**Genetic disposition** Intestinal and diffuse type (MSI stable, no EBV type)	*TP53* mutations (56%) Chromosomal instability
**Colorectal cancer**	**Epigenetic disposition** CIMP-high	Hypermethylation MSI (80%) *BRAF* mutations (53–100%) *ARID1A* mutations (39%)
	CIMP-low	*KRAS* mutations (45–92%)
	**Genetic disposition** CIMP-negative	*TP53* mutations (71–74%) Chromosomal instability
**Hepatocellular cancer**	**Epigenetic disposition** HBV, HCV infected	*ARID1A* mutations (10–17%)

### *ARID1A* mutations in the epigenetic disposition phenotype

ARID1A belongs to the SWI/SNF (SWItch/Sucrose NonFermentable) complex, whose members have ATPase activities and regulate transcription by altering chromatin structure. The SWI/SNF complex is composed of 13– 15 subunits including ARID1A, BRG1 and SNF5. The ARID1A/BRG1 complex interacts directly with p53 to effect tumor suppressor functions regulated by cell cycle-related genes [11]. Interestingly, *ARID1A* mutations are found in cancers with an epigenetic disposition, which include EBV and MSI groups in gastric cancer and the CIMP-high subtype in colorectal cancer (73–83% and 39%, respectively) [15, 24]. In gastric cancer, *ARID1A* mutations are significantly increased in *TP53* wild type as opposed to *TP53* mutated cases. Those tumors with *ARID1A* alterations tend to have prolonged, recurrence- free survival [15]. Hepatocellular carcinomas associated with hepatitis B or C virus infections exhibit *ARID1A* mutations as well as other mutations in other chromatin regulators (~50%) that are not related to *TP53* mutations [25-27]. In ovarian cancer, CCC has an epigenetic disposition with a methylation alteration subtype and *ARID1A* mutations, whereas SAC is characterized by *TP53* mutations and thus a genetic disposition. ARID1A expression is positively correlated with the expression of ER in endometrial and breast cancer [28, 29]. Both ARID1A and ER alpha expression are lost and ER pathway genes are downregulated by hypermethylation in ovarian CCC [10]. BRG1, the main ATPase of the SWI/ SNF complex, regulates chromatin remodeling during steroid hormone signaling [30]. These trends suggest that loss of chromatin remodeling complex function, including ARID1A, initiates carcinogenesis with methylation- mediated suppression of ER signaling in the inflammatory carcinogenic environment. Further exploration of the role of the SWI/SNF complex and ARID1A may indicate an even more substantial impact on the biomolecular regulation of tumors with an epigenetic disposition phenotype.

### The Warburg effect and an epigenetic disposition

In ovarian CCC, HNF1B is a fundamental molecular component of the biological characterization referred to as “ovarian CCC-likeness”. *HNF1B* expression is epigenetically regulated and its protein product is involved in glucose homeostasis. Mutations in *HNF1B* cause “RCAD syndrome” (Renal Cysts And Diabetes) and are associated with non-insulin-dependent (type 2) diabetes mellitus (NIDDM) of MODY5 (Mature-Onset Diabetes of the Young 5) as well as a syndrome of developmental renal anomalies [31]. Obesity induces impairment of glucose metabolism through silencing of *HNF1B* [32]. In ovarian CCC cells, HNF1B promotes the uptake of glucose through the glucose transporter-1 (GLUT1) protein and aerobic glycolysis, the “Warburg effect” [12]. The Warburg effect is a metabolic process in cancer that may contribute to cell survival under hypoxic conditions or in a stressful environment. Cancer cells utilize the Warburg effect to facilitate uptake and incorporation of nutrients into biomaterials (e.g., nucleotides, amino acids, and lipids) for production of a new cell [33]. Exploration of metabolic regulation specific for cancer cells is currently an area of intense research in order to identify and develop novel diagnostic tools and therapies. Recent reports support that the Warburg effect is influenced by the epigenetic regulation of genes related to glucose metabolism, such as *GLUT1*, *PK* (pyruvate kinase) and *PKM* (pyruvate kinase, muscle), in several malignancies including clear cell renal cell carcinoma, gastric cancer and colorectal cancer [34-36]. HNF1B plays a crucial role in defining an epigenetic disposition phenotype and the expression of *HNF1B* itself is regulated by DNA methylation. The Warburg effect may be epigenetically induced in cancers in the context of a stressful carcinogenic environment, leading to an epigenetic disposition phenotype.

## CONCLUSIONS

Genetic and epigenetic dispositions are proposed to characterize high-grade serous and clear cell ovarian cancers, respectively. Ovarian high-grade serous adenocarcinoma exhibits a genetic disposition with high frequency *TP53* mutations, essential for initiation of carcinogenesis, and chromosomal instability. *ARID1A* mutations often occur to initiate oncogenesis in cancers with an epigenetic disposition. An epigenetic disposition is frequent among ovarian CCC and is typified by a fundamental change in epigenetic regulation and subsequent gene expression affecting aggressive behavior leading to the development of cancer in the context of a stressful inflammatory environment. These characteristic methylation profiles may also affect cancer-specific glucose metabolism, commonly known as the Warburg effect. The concept of epigenetic and genetic dispositions is applicable for other organ malignancies, including gastric and colorectal cancers. Further exploration is needed to better understand these genetic and epigenetic disposition phenotypes across malignancies and how this can be used to further development of individualized and/ or targeted therapies.
